# PTEN Reduced UVB-Mediated Apoptosis in Retinal Pigment Epithelium Cells

**DOI:** 10.1155/2017/3681707

**Published:** 2017-02-22

**Authors:** Jia He, Chongde Long, Zixin Huang, Xin Zhou, Xielan Kuang, Lanying Liu, Huijun Liu, Yan Tang, Yuting Fan, Jie Ning, Xinqi Ma, Qingjiong Zhang, Huangxuan Shen

**Affiliations:** ^1^State Key Laboratory of Ophthalmology, Zhongshan Ophthalmic Center, Sun Yat-sen University, 54 Xianlie Road, Guangzhou 510060, China; ^2^Biobank of Eye, State Key Laboratory of Ophthalmology, Zhongshan Ophthalmic Center, Sun Yat-sen University, 54 Xianlie Road, Guangzhou 510060, China

## Abstract

Age-related macular degeneration (AMD) is a leading cause of blindness and progressive loss of central vision in the elderly population. The important factor of AMD pathogenesis is the degeneration of retinal pigment epithelial (RPE) cells by oxidative stress. Inactivation of PTEN can disrupt intercellular adhesion in the RPE cells, but the mechanism of oxidative stress is less known. Here we presented evidence that UVB-mediated oxidative stress induced apoptosis in ARPE-19 cells. Downregulation of the expression of PTEN in UVB-irradiative RPE cells triggered DNA damage and increased the level of UVB-induced apoptosis by activating p53-dependent pathway. However, overexpression of PTEN increased cell survival by suppressing p-H2A in response to DNA damage and apoptosis. When using Pifithrin-*α* (one of p53 inhibitors), the level of p53-dependent apoptosis was significantly lower than untreated, which suggested that p53 was possibly involved in PTEN-dependent apoptosis. Thus, it elucidated the molecular mechanisms of UVB-induced damage in RPE cells and may offer an alternative therapeutic target in dry AMD.

## 1. Introduction

Age-related macular degeneration (AMD) is a leading cause of blindness and progressive loss of central vision in the elderly population [1]. Two main categories of AMD exist, the dry and the wet form. Dry form is typically characterized by the accumulation of extracellular deposits (drusen) under the retina and retina pigment epithelium, thickening of Bruch's membrane, and gradual progress to late stage geographic atrophy (GA). Besides, the degeneration of retinal pigment epithelium (RPE) cells and photoreceptors is involved in choroidal neovascularization (CNV) and vascular leakage [1, 2]. Currently, clinical researches show that vascular endothelial growth factor (VEGF) can effectively treat wet AMD [3, 4]. However, little is known about the mechanisms underlying dry AMD. It is, therefore, urgent to find ways to know effective therapeutic method to delay the progression of dry AMD.

RPE cells play a vital role in the absorption of light energy and transport of metabolites and nutrients between photoreceptors and choroidal capillaries [5]. It is accepted that degeneration of RPE cells is an important causative factor of AMD pathogenesis. Similar to other age-related diseases, the pathogenesis of AMD is complex and multifactorial, including genetic, environmental, dietary, and behavioural factors and solar irradiation [6–9]. Because of the ozone layer depletion, the excessive exposure to solar ultraviolet (UV) radiation especially UVB is mainly absorbed by the cornea and lens and a fraction reaches the retina [10]. Long-time UVB irradiation is considered as an important cause of AMD [11–13]. These factors induce chronic inflammatory processes and oxidative stress, which ultimately leads to retinal damage and degradation of sensitive photoreceptor cells. However, the mechanisms of UVB-induced retinal phototoxicity remain unclear.

It is an essential role for PTEN (phosphatase and tensin homolog) in multiple biological processes, including the regulation of genomic instability, DNA repair, cellular senescence, and cell migration, besides a well-characterized tumor suppressor [14, 15]. It has been revealed that PTEN plays a key function for DNA repair and viability following DNA-damaging UVB radiation [16]. In addition, previous report showed that PTEN knockout mice lead to hypertrophic optic nerve [17]. Furthermore, recent studies have reported that inactivation of PTEN significantly disrupts intercellular adhesion in the RPE, which leads to AMD-like retinal degeneration in mice [18]. However, the function of PTEN and DNA damage following exposure to UVB remains obscure in RPE cells.

In this study, we aimed to investigate possible mechanisms involved in UVB-induced retinal damage and function of PTEN.

## 2. Materials and Methods

### 2.1. Cell Culture and Reagents

Human RPE cell line (ARPE-19) was obtained from the American Type Culture Collection (ATCC). Cell was cultured in DMEM growth medium (HyClone, GE Healthcare Life Science, USA) supplemented with 10% FBS (Gibco, Life Technology, USA), penicillin (100 *μ*g/mL), and streptomycin (100 *μ*g/mL) in a 5% CO_2_ humidified atmosphere at 37°C. Pifithrin-*α* was purchased from Sigma-Aldrich (USA).

### 2.2. UVB Irradiation

For UVB irradiation, the medium was removed and the cells were covered with phosphate-buffered saline (PBS) and exposed to a germicidal 8 Watt UV lamp (305 nm) at fixed distance and variable doses. After UVB irradiation, cells were cultured in fresh medium for 24 hours. The UV lamp could be measured by mJ/cm^2^ for the UVB radiation.

### 2.3. Intracellular Reactive Oxidative Species (ROS) and Apoptosis Measurement

ROS was examined using dihydroethidium (DHE; Millipore, EMD Millipore Corporation, Hayward, CA), according to the manufacturer's instructions. Briefly, RPE cells were seeded into 60 mm culture plates and split using 0.25% trypsin and washed with PBS. After gathering cells, DHE was added to each dish for 30 min at 37°C in dark. And then, the fluorescence intensity was measured using a Muse™ Cell Analyze. The percentages of apoptosis were examined using Multicaspase Kit (Millipore). After gathering cells, Multicaspase was added to each dish for 30 min at 37°C in dark and incubated with 7-aminoactinomycin D (7-AAD, Millipore) for 5 min at room temperature. The events for live, dead early, and late apoptotic cells were counted with the Muse Cell Analyzer.

### 2.4. Cell Viability

The 3-(4, 5-dimethylthiazol-2-yl)-2,5-diphenyl-tetrazolium bromide (MTT) assay was used to detect cell viability. Briefly, ARPE-19 cells were seeded in 96-well plates. After different time of culture, cells were incubated with 20 *μ*L MTT (5 mg/mL, Sigma-Aldrich) for 4 h at 37°C. After removal of the medium, 150 *μ*L dimethyl sulfoxide (DMSO) solution was added to dissolve formazan crystals. The absorbance at 490 nm wavelengths was measured by a microplate reader (BioTek Instruments, Winooski, VT).

### 2.5. Reverse Transcription and Real-Time Polymerase Chain Reaction

Real-time RT-PCR was performed as described previously [19]. Total RNA was extracted by using the Trizol Reagent (Invitrogen, Carlsbad, CA). By using the PrimeScriptII 1st Strand cDNA Synthesis Kit (TaKaRa, Japan), the total RNA was subject to reverse transcription. The RT-PCR analysis was performed in LightCycler 96 (Roche, Basel, Switzerland) by using the SYBR Premix Ex TaqTM Kit (TaKaRa). The human* GAPDH* gene was used as an endogenous control for sample normalization. The primers used for human* PTEN* are as follows: forward 5′-CGAACTGGTGTAATGATATGT-3′ and reverse 5′-CATGAACTTGTCTTCCCGT-3′; GAPDH forward 5′-GAGTCAACGGATTTGGTCGT-3′ and reward 5′-GACAAGCTTCCCGTTCTCAG-3′.

### 2.6. Western Blotting

Western blotting was performed as described previously [20]. Those primary antibodies were PTEN (Cell Signaling Technology, Danvers, MA), p53 (Cell Signaling Technology, Danvers, MA), p-ATM (Santa Cruz, Santa Cruz Biotechnology lnc, CA), p-H2A (Santa Cruz, Santa Cruz Biotechnology lnc, CA), and *α*-Tubulin (Affinity, USA). *α*-Tubulin was used as an internal control. The intensities of band were quantified by using Image J software.

### 2.7. Plasmids and Transfection

The following small hairpin RNA (shRNA) lentiviral constructs targeting the* PTEN* (Genbank Number NM_000314.6) genes were obtained from Thermo Scientific. The hairpin sequence numbers are TRCN0000002745-9. A vector for mammalian overexpression of PTEN was generated by cloning* PTEN* CDS into the multiple cloning sites (MCS) of the pOZ-FH-N vector via* Xho*I and* BamH*I restriction sites. Integrity of the construct was verified by sequencing.

As described in previous study [21], 50% confluency 293T cells seeded in 60 mm plates were transfected with lentiviral particles containing the PTEN shRNA and overexpression PTEN by using Effectene Transfection Reagent (Qiagen, Hilden, Germany). RPE cells were plated at 50% confluence in 60 mm plates and subsequently infected with viral supernatant three times containing 2 *μ*g/mL Polybrene (Sigma-Aldrich, St. Louis, MO). Then, cell culture medium was replaced with complete medium with puromycin (1.5 *μ*g/mL, Sigma-Aldrich) for three days.

### 2.8. Statistical Analysis

All assays are performed in triplicate. These results are expressed as the mean ± standard deviation (SD). Statistical analyses are performed with Student's *t*-test for comparison of two groups (Graph prism5 software). Significant* P* values are shown as *P* < 0.05 (*∗*), *P* < 0.01 (*∗∗*), and *P* > 0.05 (#).

## 3. Results

### 3.1. UVB Irradiation Induced Reactive Oxygen Species (ROS) and Increased Apoptosis and Downregulated PTEN Expression in RPE Cells

**Figure 1:**
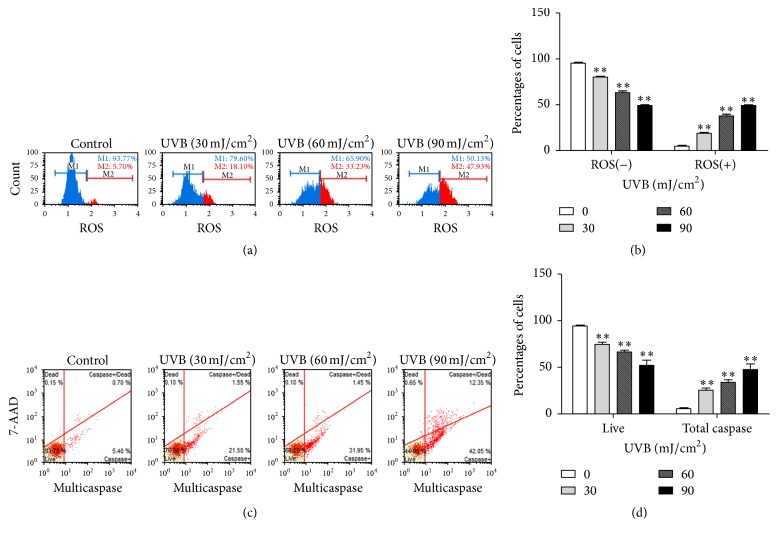
UVB irradiation induced reactive oxygen species (ROS) and increased apoptosis. RPE cells were exposed to 0, 30, 60, and 90 mJ/cm^2^ of UVB (305 nm) and then incubated for 24 h. (a) Intracellular ROS was determined by flow cytometry. (b) Bar graphs represent the percentage of RPE cells in control or UVB treatment group. (c) The untreated group was regarded as the control group. The percentage of live and apoptosis positive cells was showed. (d) Bar graphs represent the percentage of RPE cells in control or UVB treatment group (^*∗∗*^*P* < 0.01,* t* test, *n* = 3, bars represent SD).

As shown in Figures 1(a) and 1(b), after 24 h following UVB irradiation, the percentages of ROS positive cells were clear elevation in a dose-dependent manner. It was indicated that UVB radiation greatly increased ROS production. To determine the effect of UVB radiation in ARPE-19 cells, the percentage of apoptosis was detected by flow cytometry. After 24 h following UVB treatment, it was found that the percentage of apoptosis was increased in a dose-dependent pattern (Figures 1(c) and 1(d)). In addition, the experimental results showed that RPE cells induced apoptosis approximately 30% when treated with high dose UVB (32% apoptosis at 60 mJ/cm^2^, 55% at 90 mJ/cm^2^). Thus, two doses (60, 90 mJ/cm^2^) were chosen for the following experiments. These results suggested that UVB-mediated oxidative stress could induce apoptosis in RPE cells.

**Figure 2:**
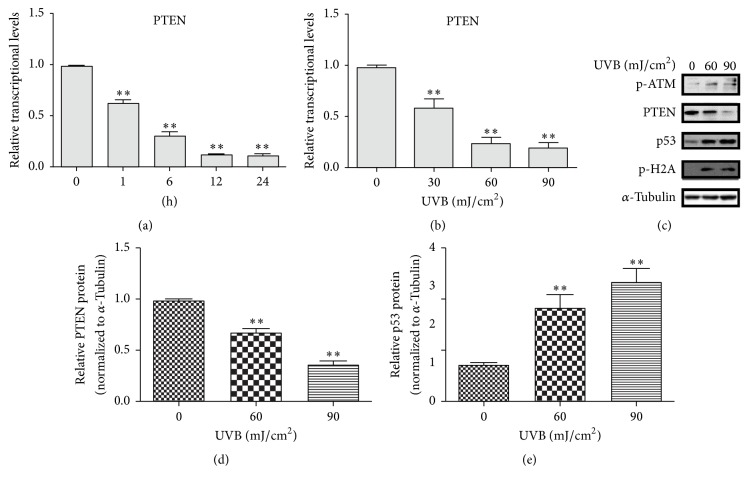
UVB irradiation downregulated PTEN expression and induced DNA damage in RPE cells. (a) Cells were exposed to UVB radiation (60 mJ/cm^2^) and then incubated for 0–24 h. (b) Cells were exposed to 0–90 mJ/cm^2^ UVB and then incubated for 24 h. mRNA expression level of PTEN was analyzed by Real-Time PCR. The relative expression data were presented after normalization to GAPDH. (c) Following UVB exposure (0, 60, 90 mJ/cm^2^) in RPE cells for 24 hours. Protein levels were detected by western blot. *α*-Tubulin expression was detected as the loading control. (d, e) Bar graphs represent the relative PTEN and p53 protein levels. The ratio of PTEN was quantized by using Image J (^*∗∗*^*P* < 0.01,* t* test, *n* = 3, bars represent SD).

Previous report indicated that inactivation of PTEN induced AMD-like retinal degeneration in mice, which led us to further study the role of PTEN upon UVB irradiation in RPE cells. Thus, we analyzed the mRNA expression levels of* PTEN* by Real-Time PCR. RT-PCR analysis showed a lower expression level of* PTEN* mRNA in a dose- and time-dependent manner by UVB irradiation than the nonradiated group, respectively. As shown in Figures 2(a) and 2(b), after different time (0, 1, 6, 12, and 24 h) and various concentration (0, 30, 60, and 90 mJ/cm^2^) following UVB irradiation, an obvious decrease of* PTEN* expression was observed. To further explore the molecular mechanism of UVB-induced retinal damage, we investigated the several DNA damage- and apoptosis-related proteins, including p-ATM, p-H2A, and p53. Consistent with mRNA result, western blot indicated that UVB treatment resulted in a clear decrease PTEN expression in a dose-dependent manner (Figures 2(c) and 2(d)). However, p-ATM, p-H2A, and p53 were elevated in a dose-dependent manner after 24 h UVB exposure (Figures 2(c) and 2(e)). In summary, UVB irradiation induced high levels of oxidative stress and apoptosis in RPE cells, resulting in decreased expression of PTEN and subsequent DNA damage related to p53 activation.

### 3.2. Inhibiting PTEN Enhanced UVB-Induced Apoptosis and Activation of p53 in RPE Cells

**Figure 3:**
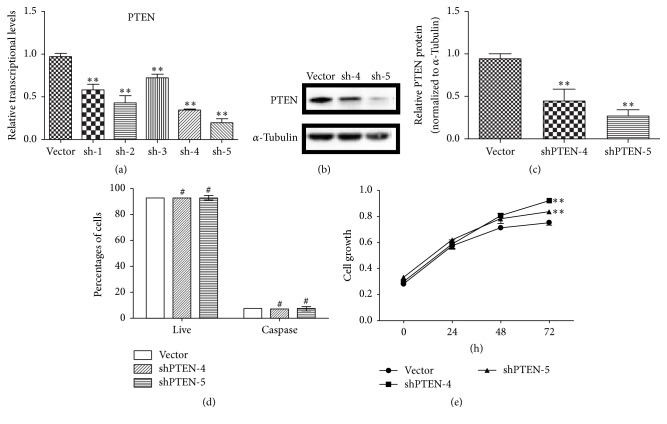
Inhibiting PTEN could promote RPE cells growth but had no impacts on cell apoptosis. RPE cells were stably transfected with PTEN shRNA plasmids. (a) The knockdown efficiency of PTEN was measured by Real-Time PCR. (b) Western blots were conducted to detect the PTEN protein expression level and use *α*-Tubulin as an internal control. (c) Bar graphs represent the relative PTEN protein levels. (d) Apoptosis of cells was quantified by Multicaspase/7-AAD stain and analyzed by flow cytometry. (e) Cell viability was detected by MTT assay. The results are expressed as mean ± SEM from at least 3 experiments (^*∗∗*^*P* < 0.01, ^#^*P* > 0.05,* t* test, *n* = 3, bars represent SD).

Given the essential role of PTEN in maintaining genomic instability DNA repair and cell death, we thus explored whether PTEN was a necessary protein in DNA damage induced apoptosis RPE cells. To this end, the expression of* PTEN* was inhibited by lentiviral-based small hairpin RNA (shRNA). Five* PTEN* shRNA plasmids were transfected into RPE cells. Then, the transfection and expression efficiency were determined by Real-Time PCR (Figure 3(a)). The mRNA expression levels of* PTEN* were remarkably inhibited in two of five stable shRNA PTEN RPE cell lines. Thus, those high efficiency cells (shRNA PTEN-4 and shRNA PTEN-5) were chosen for the following experiments (Figures 3(b) and 3(c)). In two stable PTEN knockdown cell lines, apoptosis of RPE cells had no significant change (Figure 3(d)); however the cell proliferation had a slight augment (Figure 3(e)). These results indicated that knockdown the expression of PTEN in RPE cells could affect growth but had no impacts on cell apoptosis.

**Figure 4:**
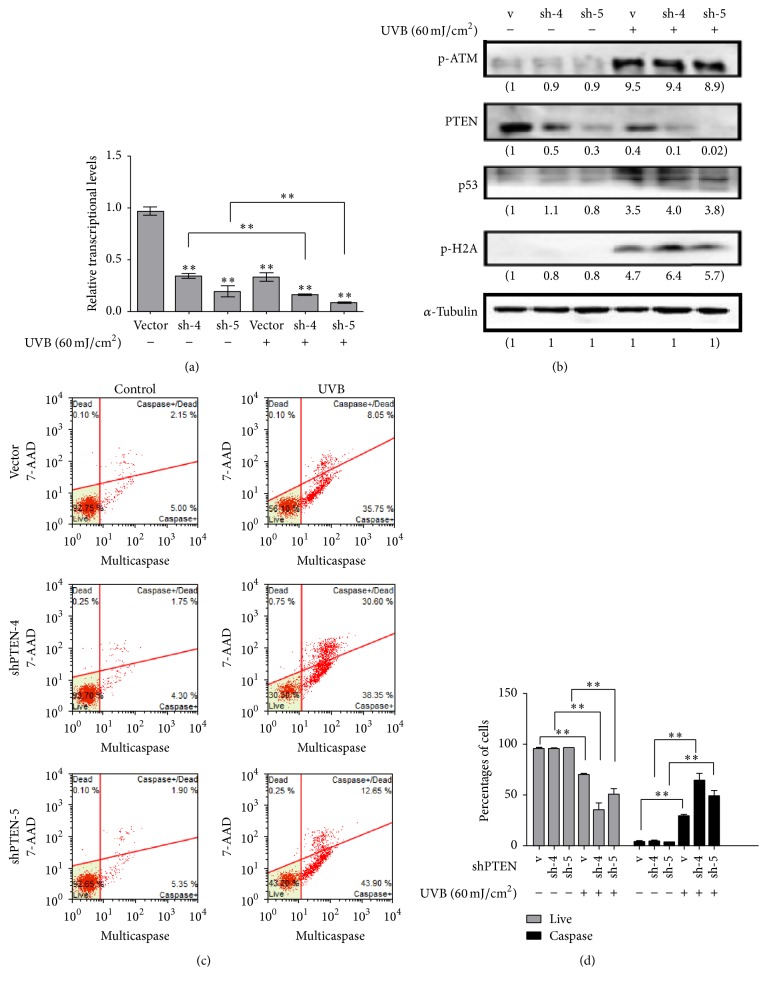
Inhibiting PTEN enhanced levels of UVB-induced apoptosis and activation of p53 in RPE cells. The vector and inhibiting PTEN cells were irradiated with UVB (60 mJ/cm^2^) and then incubated for 24 h. “v” represented vector; “sh-4 and sh-5” represented sh-PTEN4 and sh-PTEN5. (a) The relative mRNA expression level of PTEN was determined in RPE cells by Real-Time PCR. The result was normalized to GAPDH. (b) The expression levels of proteins were analyzed by western blot. (c) Apoptosis of cells was measured by flow cytometry. (d) Data are represented as the mean ± SD of three independent experiments (^*∗∗*^*P* < 0.01,* t* test).

To further explore the function of PTEN under UVB treatment, we exposed three RPE cells in 60 mJ/cm^2^ dose for 24 h and then detected the apoptosis level. As shown in Figure 4(a), UVB treatment decreased the mRNA expression of* PTEN*; moreover, the expression level of* PTEN* was clearly lower in those two knockdown cell lines than vector control. In accord with the Real-Time PCR result, western analysis indicated that PTEN protein level was significantly decreased in PTEN knockdown cell lines (Figure 4(b)). In addition, western result also suggested that p-H2A and p53 protein levels were significantly increased in those PTEN inhibiting groups compared with the vector control group, whereas p-ATM was still keeping with vector control (Figure 4(b)). The percentage of apoptosis positive cells was markedly increased in those two RPE cell lines (Figures 4(c) and 4(d)). The data above was indicated reducing PTEN expression and increasing p53 may be the cause of apoptosis in those PTEN knockdown cells.

### 3.3. Overexpression of PTEN Decreased Apoptosis and DNA Damage by UVB Irradiation in RPE Cells

**Figure 5:**
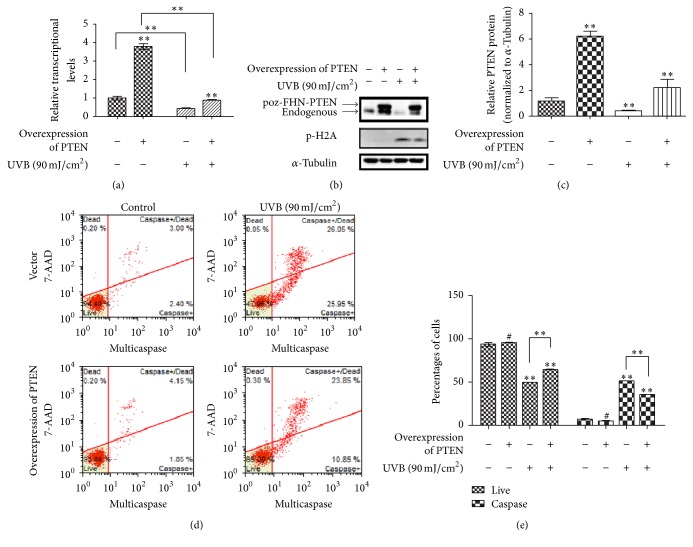
The overexpression of PTEN attenuated cell apoptosis and DNA damage induced by UVB irradiation. RPE cells were stably transfected with overexpression plasmid. Then cells were treated with UVB (90 mJ/cm^2^) for 24 h. Levels of the mRNA (a) and protein (b) expression were detected by Real-Time PCR and western blot. And DNA damage related protein p-H2A was detected. (c) Bar graphs represent the relative PTEN protein levels normalized to that of *α*-Tubulin of different groups. (d) Cells were stained with Multicaspase/7-AAD for quantitative measurement using flow cytometry. (e) A representative apoptosis along with the statistical data was shown. These results are expressed as mean ± SD from three experiments (^*∗∗*^*P* < 0.01, ^#^*P* > 0.05 versus control).

In keeping with previous result, knockdown PTEN increased apoptosis of RPE cells upon UVB exposure. Next, the overexpression system was used. RPE cells were stably transfected with the pOZ-FHN-PTEN plasmid. It was observed that total mRNA expression of* PTEN* was elevated about three to four times, comparing to the endogenous* PTEN* level. UVB treatment decreased the mRNA expression of* PTEN*, but the decrease pattern was attenuated when the cells were overexpressed PTEN (Figure 5(a)). And the result was confirmed by western blot. As shown in Figures 5(b) and 5(c), comparing to the control cells, PTEN protein decrease induced by UVB was partially attenuated in PTEN overexpression cells, mainly on the level of endogenous PTEN. In addition, compared to vector, we found a clear decrease of p-H2A level in overexpression PTEN cell following UVB exposure (Figure 5(b)). By using flow cytometry, we examined percentage of apoptosis with UVB (90 mJ/cm^2^) treatment and found in overexpression PTEN cells apoptosis was less than empty control cells (Figures 5(d) and 5(e)). In summary, these results strongly suggested that overexpression PTEN could reduce oxidative stress, protect cells from DNA damage, inactive form of p-H2A, and decrease apoptosis.

### 3.4. P53 Inhibitor Decreased UVB-Induced Apoptosis

**Figure 6:**
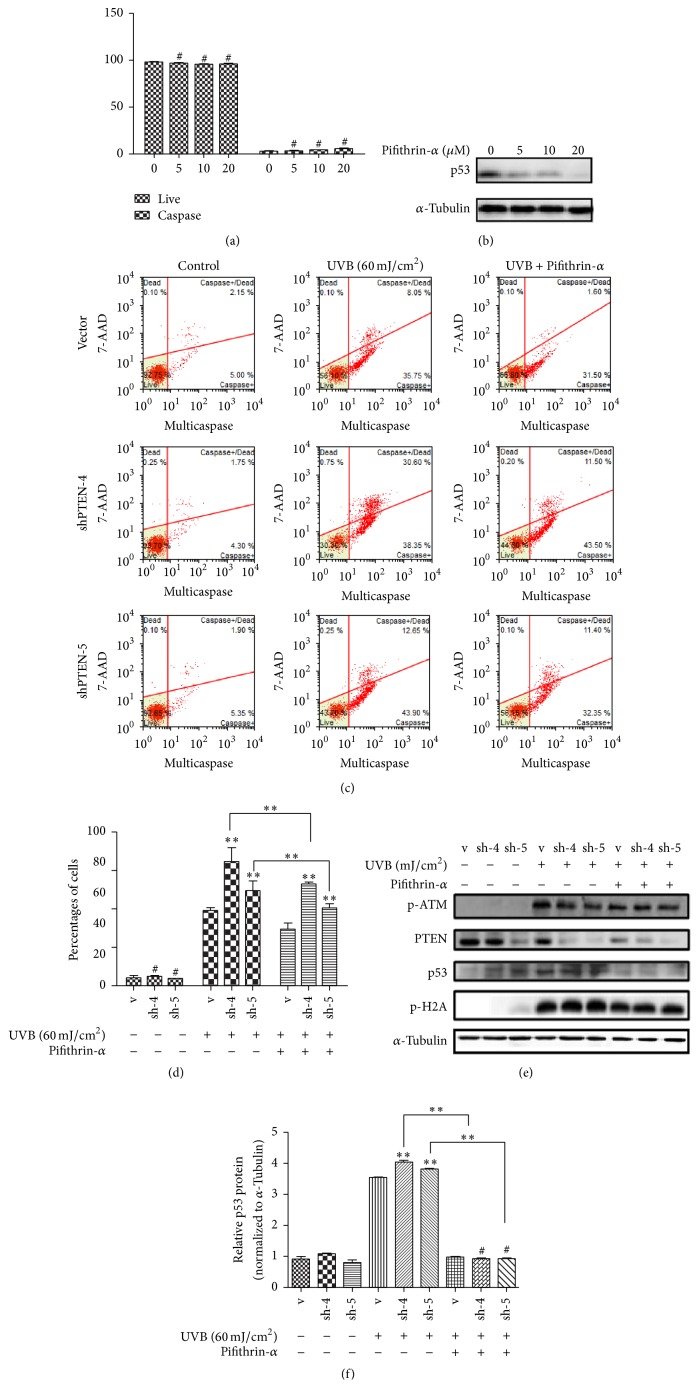
p53 inhibitor decreased UVB-induced apoptosis. RPE cells were pretreated with p53 inhibitor Pifithrin-*α* (10 *μ*M) for 1 h and then incubated for 24 h. (a) Cells were stained with Multicaspase/7-AAD for quantitative measurement using flow cytometry. (b) Western blots were conducted to detect p53 protein expression level and use *α*-Tubulin as an internal control. RPE cells were pretreated with p53 inhibitor Pifithrin-*α* (10 *μ*M) for 1 h and these cells were exposed to UVB (60 mJ/cm^2^) and then incubated for 24 h. “v” represented vector and “sh-4 and sh-5” represented sh-PTEN4 and sh-PTEN5. (c) Cells were stained with Multicaspase/7-AAD for quantitative measurement using flow cytometry. (d) A representative apoptosis along with the statistical data was shown. (e) Western blots were conducted to detect expression levels of protein and use *α*-Tubulin as a loading control. (f) Using Image J quantized the ratio of p53. These data are shown as the mean ± SD of three independent experiments (^*∗∗*^*P* < 0.01, ^#^*P* > 0.05,* t *test).

Existing data implied that p53 was markedly activated following UVB exposure in RPE and stable shRNA PTEN cell lines. Next, we explored whether PTEN could affect apoptosis through p53. It was well known that Pifithrin-*α* was a reversible inhibitor of p53-mediated apoptosis. We Treated RPE cell lines with various dose Pifithrin-*α* (0, 5, 10, 20 *μ*M) for 24 h; the percentages of apoptosis make no difference (Figure 6(a)). However, expression level of p53 protein was decreased in dose-dependent manner (Figure 6(b)).

As expected, we treated those three cell lines with Pifithrin-*α* (10 *μ*M) for 1 h before exposure to UVB (60 mJ/cm^2^). The level of apoptosis that treated with Pifithrin-*α* group was significantly lower than the untreated (Figures 6(c) and 6(d)). Furthermore, western results indicated that p53 protein level was significantly decreased in the Pifithrin-*α* treatment group (Figures 6(e) and 6(f)). However, compared to untreated Pifithrin-*α* groups, the expressions of PTEN, p-ATM, and p-H2A (Figure 6(e)) in the treatment groups had no significant change. Thus, these results collectively showed that addition of Pifithrin-*α* did not alter apoptosis in the control cell lines but did decrease apoptosis of the UVB-irradiated cells. Those results also suggested that PTEN mediate p53-induced apoptosis.

## 4. Discussion

This study showed for the first time that UVB triggers the PTEN pathway in RPE cells to cause cell damage and apoptosis. These results may demonstrate the mechanisms of UVB-induced retinal damage. Overexpression PTEN protected against UVB-induced apoptosis by suppressing p-H2A. This study provides assumption for the idea that upregulated PTEN may help individuals in decreasing retinal oxidative stress.

Previous study and reports showed that UVB could induce oxidative stress in RPE cells, which play a role in providing photoreceptor functions and structural support to the retina [5]. It had been accepted that degeneration of RPE cells was an important causative factor of AMD pathogenesis. PTEN was a well-characterized tumor suppressor [14]. PTEN also was involved in multiple biological processes, including the regulation of genomic instability, DNA repair, cellular senescence, and cell migration. Moreover, PTEN positively regulates UVB-induced DNA damage repair in cells [16]. In DNA damage response (DDR) pathways, ATM-Chk2 are activated in oxidative stress to coordinate DNA repair, cell cycle progression, transcription, apoptosis, and senescence [22]. It has been demonstrated that ATM can promote the survival of PTEN-deficient cells through the signaling of oxidative DNA damage [23]. Moreover, accumulating evidence indicates that p53 play an important role in UVB-induced DNA damage in Human HaCaT Keratinocytes cells [24]. And UVB dose-dependently suppressed the growth of RPE cells by activating the phosphatidylinositol 3-kinase (PI3K) pathway [25]. Considering UV irradiation in corneal epithelium, Pifithrin-*α* could inhibit p53 and decrease damaged cells and apoptosis [26]. Thus, DNA damage related protein p-ATM and induced apoptosis p53 were estimated in the following experiment to regulate PTEN interaction.

In this study, we found that PTEN is decreased following UVB exposure in a dose- and time-dependent manner. In addition, we also found that p-ATM and p-H2A and p53 were clearly activated when PTEN was suppressed. These results suggested UVB-induced apoptosis was involved in PTEN and DNA damage related protein. Previous report showed that an aberrant interplay between the redox-sensitive PTEN and PI3k/Akt signaling in rostral ventrolateral medulla and neurogenic hypertension in spontaneously hypertensive rats [27]. Importantly, additional studies revealed that inactivation of PTEN significantly disrupted intercellular adhesion in the RPE and PTEN by activating PI3K signal pathway response to oxidative stress [28].

However, the function of PTEN has not been well-characterized in the RPE cells following UVB irradiation. To further illustrate the role of PTEN, we transfected five stable shRNA PTEN plasmids into RPE cells and two high efficiency cell lines were chosen for the following experiments. Following UVB exposure, expressions of two stable shRNA PTEN cells mRNA and protein were strikingly reduced which compared with empty vector. Importantly, the decreased shRNA PTEN expression was accompanied by DNA damage dependent activation of p-H2A and p53 protein. Furthermore, apoptosis level was significantly increased upon UVB treatment in shRNA PTEN cells. These results indicated that UVB caused DNA damage and apoptosis of RPE cells and this process could be elevated by inhibiting of PTEN. This may be due to PTEN signals playing an important role in RPE cells following UVB irradiation. Moreover, the apoptosis of RPE cells was significantly decreased after PTEN overexpression upon UVB exposure. Consistently, p-H2A level was downregulated. Likewise, in previous reports employing PTEN overexpression, a reduction of DNA damage and apoptosis of HELA cells was demonstrated [29]. In addition, it has been reported that p53 pathway was activated following UVB exposure in RPE cells. These results are consistent with those of our study. Furthermore, our data showed that these vector and shRNA PTEN cells had no significant difference change in PTEN mRNA expression level in response to Pifithrin-*α* treatment after UVB irradiation. Given these study results, we draw a conclusion that apoptotic effect of PTEN might be due to p53 activation. Moreover, inhibiting PTEN cell lines exhibited higher p53 activity and more tended to apoptosis.

There are many potential mechanisms of PTEN signaling under UVB irradiation. It is of interesting consequences in this paper. For example, although shPTEN-5 transfection and expression efficiency were higher than shPTEN-4, the percentages of apoptosis were lower (Figure 4). This suggested that the expression of endogenous PTEN might have no direct relationship with the extent of apoptosis. Besides, endogenous PTEN level increases when cells were overexpressed PTEN. In addition, it was mainly the endogenous PTEN protein that decreased in PTEN overexpression cell lines after UVB exposure (Figure 5). Above all, further mechanistic studies are needed in order to understand the precise molecular mechanisms.

In the elderly population, age-related macular degeneration (AMD) is a leading cause of blindness [30]. Although it has been reported that VEGF can effectively treat wet AMD [3], little is known about the mechanisms and therapeutic method. Our study indicated the selective sensitivity of upregulated PTEN or p53 inhibition could reduce UVB-induced apoptosis in vivo. It suggests that this maybe presents a novel approach to targeted dry AMD therapy in the clinic.

## 5. Conclusions

In summary, we have focused on the possible mechanisms involved in UVB-induced retinal damage. In PRE cells, UVB irradiation induced ROS, increased apoptosis, downregulated the expression of PTEN, and upregulated p53 expression. Inhibiting PTEN expression enhanced the levels of UVB-induced apoptosis and activation of p53. Furthermore, overexpression PTEN and p53 inhibitor protected PRE cells against UVB-induced apoptosis. Targets on PTEN pathway could provide one strategy, which may prove therapeutically beneficial for dry AMD treatment.
